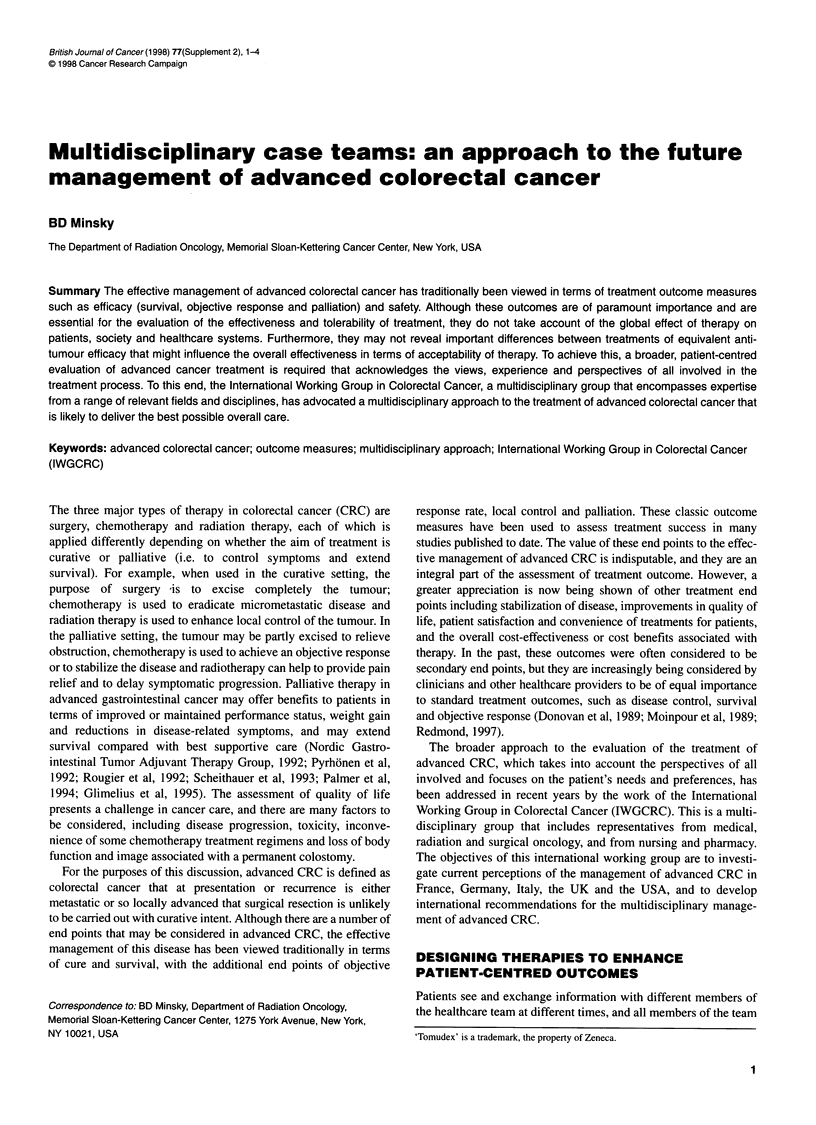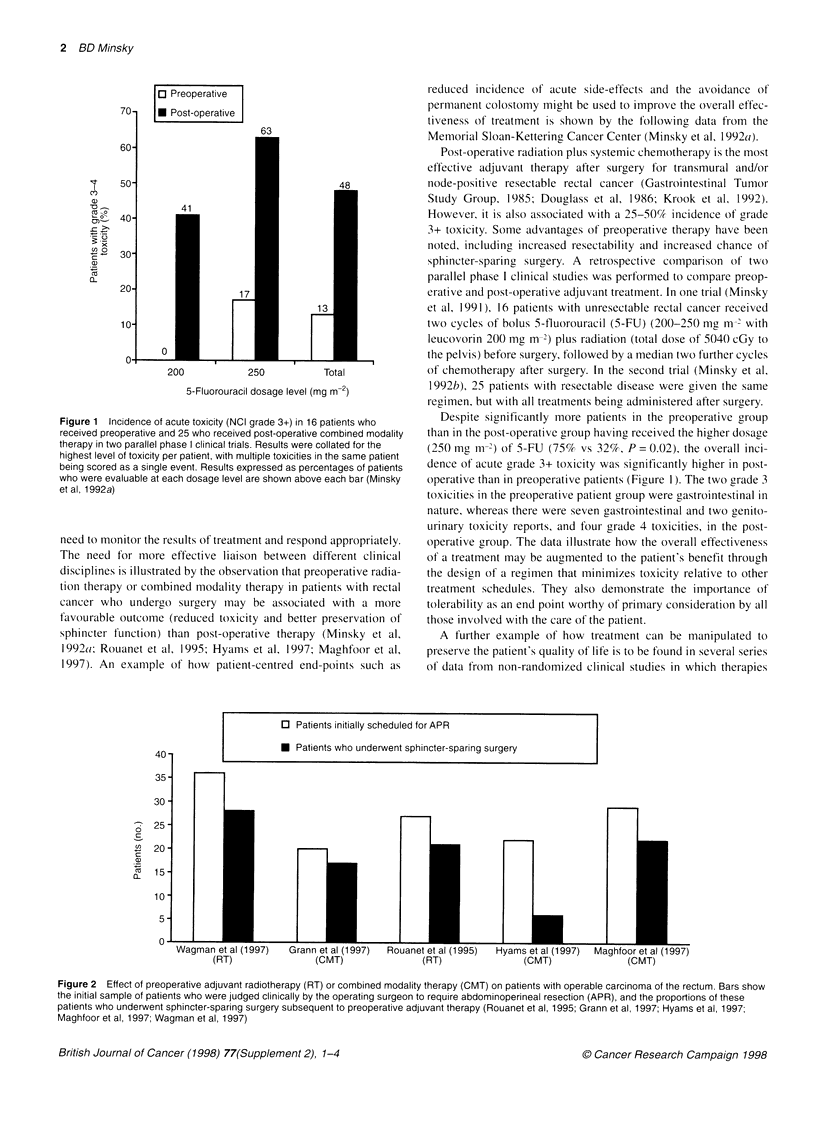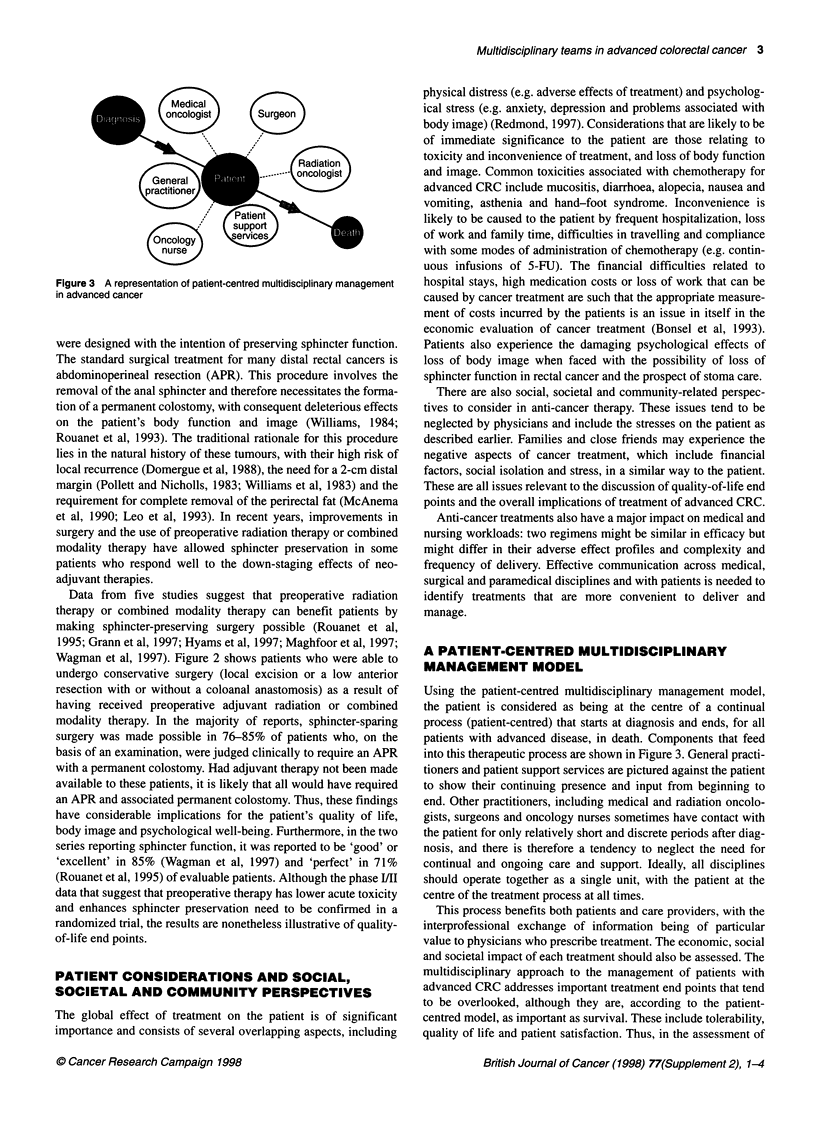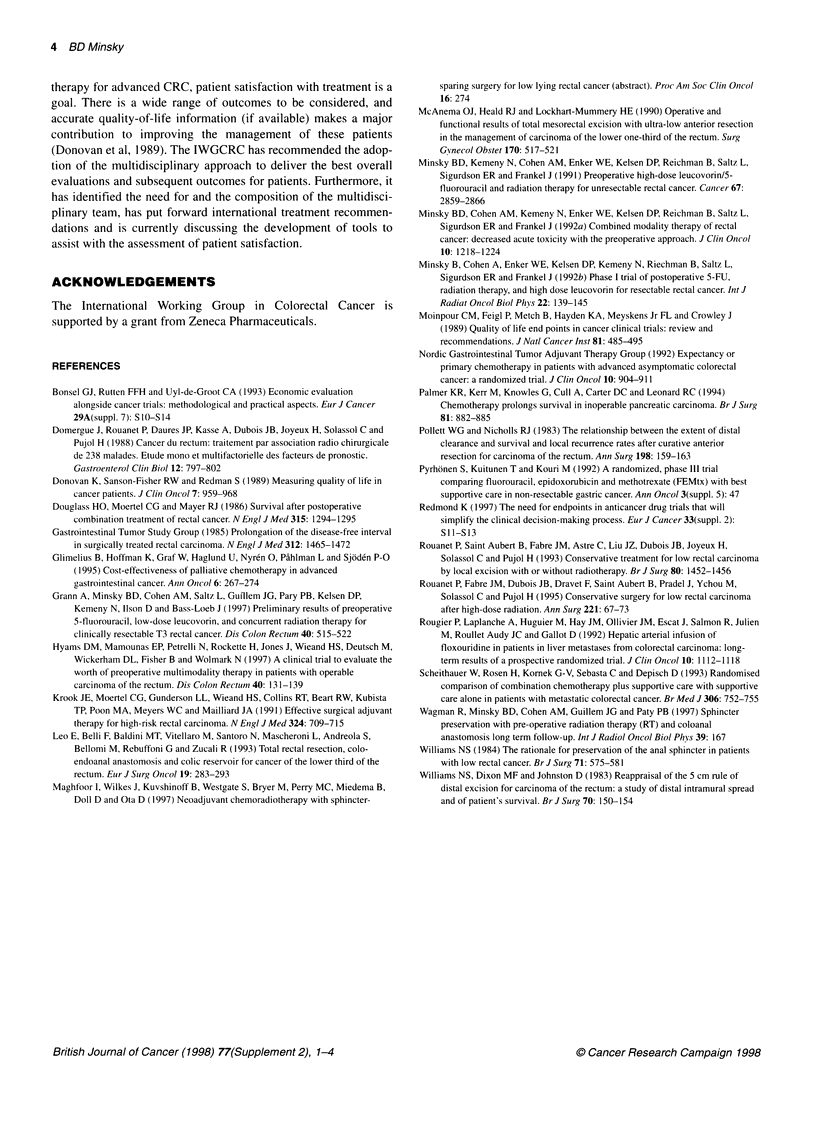# Multidisciplinary case teams: an approach to the future management of advanced colorectal cancer.

**DOI:** 10.1038/bjc.1998.418

**Published:** 1998

**Authors:** B. D. Minsky

**Affiliations:** The Department of Radiation Oncology, Memorial Sloan-Kettering Cancer Center, New York, NY 10021, USA.

## Abstract

The effective management of advanced colorectal cancer has traditionally been viewed in terms of treatment outcome measures such as efficacy (survival, objective response and palliation) and safety. Although these outcomes are of paramount importance and are essential for the evaluation of the effectiveness and tolerability of treatment, they do not take account of the global effect of therapy on patients, society and healthcare systems. Furthermore, they may not reveal important differences between treatments of equivalent anti-tumour efficacy that might influence the overall effectiveness in terms of acceptability of therapy. To achieve this, a broader, patient-centred evaluation of advanced cancer treatment is required that acknowledges the views, experience and perspectives of all involved in the treatment process. To this end, the International Working Group in Colorectal Cancer, a multidisciplinary group that encompasses expertise from a range of relevant fields and disciplines, has advocated a multidisciplinary approach to the treatment of advanced colorectal cancer that is likely to deliver the best possible overall care.


British Journal of Cancer (1998) 77(Supplement 2), 1–4
© 1998 Cancer Research Campaign

### Multidisciplinary case teams: an approach to the future management of advanced colorectal cancer

**BD Minsky**

The Department of Radiation Oncology, Memorial Sloan-Kettering Cancer Center, New York, USA

**Summary** The effective management of advanced colorectal cancer has traditionally been viewed in terms of treatment outcome measures such as efficacy (survival, objective response and palliation) and safety. Although these outcomes are of paramount importance and are essential for the evaluation of the effectiveness and tolerability of treatment, they do not take account of the global effect of therapy on patients, society and healthcare systems. Furthermore, they may not reveal important differences between treatments of equivalent anti-tumour efficacy that might influence the overall effectiveness in terms of acceptability of therapy. To achieve this, a broader, patient-centred evaluation of advanced cancer treatment is required that acknowledges the views, experience and perspectives of all involved in the treatment process. To this end, the International Working Group in Colorectal Cancer, a multidisciplinary group that encompasses expertise from a range of relevant fields and disciplines, has advocated a multidisciplinary approach to the treatment of advanced colorectal cancer that is likely to deliver the best possible overall care.

**Keywords:** advanced colorectal cancer; outcome measures; multidisciplinary approach; International Working Group in Colorectal Cancer (IWGCRC)

The three major types of therapy in colorectal cancer (CRC) are surgery, chemotherapy and radiation therapy, each of which is applied differently depending on whether the aim of treatment is curative or palliative (i.e. to control symptoms and extend survival). For example, when used in the curative setting, the purpose of surgery is to excise completely the tumour; chemotherapy is used to eradicate micrometastatic disease and radiation therapy is used to enhance local control of the tumour. In the palliative setting, the tumour may be partly excised to relieve obstruction, chemotherapy is used to achieve an objective response or to stabilize the disease and radiotherapy can help to provide pain relief and to delay symptomatic progression. Palliative therapy in advanced gastrointestinal cancer may offer benefits to patients in terms of improved or maintained performance status, weight gain and reductions in disease-related symptoms, and may extend survival compared with best supportive care (Nordic Gastro-intestinal Tumor Adjuvant Therapy Group, 1992; Pyrhönen et al, 1992; Rougier et al, 1992; Scheithauer et al, 1993; Palmer et al, 1994; Glimelius et al, 1995). The assessment of quality of life presents a challenge in cancer care, and there are many factors to be considered, including disease progression, toxicity, inconvenience of some chemotherapy treatment regimens and loss of body function and image associated with a permanent colostomy.

For the purposes of this discussion, advanced CRC is defined as colorectal cancer that at presentation or recurrence is either metastatic or so locally advanced that surgical resection is unlikely to be carried out with curative intent. Although there are a number of end points that may be considered in advanced CRC, the effective management of this disease has been viewed traditionally in terms of cure and survival, with the additional end points of objective response rate, local control and palliation. These classic outcome measures have been used to assess treatment success in many studies published to date. The value of these end points to the effective management of advanced CRC is indisputable, and they are an integral part of the assessment of treatment outcome. However, a greater appreciation is now being shown of other treatment end points including stabilization of disease, improvements in quality of life, patient satisfaction and convenience of treatments for patients, and the overall cost-effectiveness or cost benefits associated with therapy. In the past, these outcomes were often considered to be secondary end points, but they are increasingly being considered by clinicians and other healthcare providers to be of equal importance to standard treatment outcomes, such as disease control, survival and objective response (Donovan et al, 1989; Moinpour et al, 1989; Redmond, 1997).

The broader approach to the evaluation of the treatment of advanced CRC, which takes into account the perspectives of all involved and focuses on the patient's needs and preferences, has been addressed in recent years by the work of the International Working Group in Colorectal Cancer (IWGCRC). This is a multidisciplinary group that includes representatives from medical, radiation and surgical oncology, and from nursing and pharmacy. The objectives of this international working group are to investigate current perceptions of the management of advanced CRC in France, Germany, Italy, the UK and the USA, and to develop international recommendations for the multidisciplinary management of advanced CRC.

### DESIGNING THERAPIES TO ENHANCE PATIENT-CENTRED OUTCOMES

Patients see and exchange information with different members of the healthcare team at different times, and all members of the team

*Correspondence to:* BD Minsky, Department of Radiation Oncology, Memorial Sloan-Kettering Cancer Center, 1275 York Avenue, New York, NY 10021, USA

'Tomudex' is a trademark, the property of Zeneca.

**Figure 1** Incidence of acute toxicity (NCI grade 3+) in 16 patients who received preoperative and 25 who received post-operative combined modality therapy in two parallel phase I clinical trials. Results were collated for the highest level of toxicity per patient, with multiple toxicities in the same patient being scored as a single event. Results expressed as percentages of patients who were evaluable at each dosage level are shown above each bar (Minsky et al, 1992*a*)

need to monitor the results of treatment and respond appropriately. The need for more effective liaison between different clinical disciplines is illustrated by the observation that preoperative radiation therapy or combined modality therapy in patients with rectal cancer who undergo surgery may be associated with a more favourable outcome (reduced toxicity and better preservation of sphincter function) than post-operative therapy (Minsky et al, 1992*a*; Rouanet et al, 1995; Hyams et al, 1997; Maghfoor et al, 1997). An example of how patient-centred end-points such as reduced incidence of acute side-effects and the avoidance of permanent colostomy might be used to improve the overall effectiveness of treatment is shown by the following data from the Memorial Sloan-Kettering Cancer Center (Minsky et al, 1992*a*).

Post-operative radiation plus systemic chemotherapy is the most effective adjuvant therapy after surgery for transmural and/or node-positive resectable rectal cancer (Gastrointestinal Tumor Study Group, 1985; Douglass et al, 1986; Krook et al, 1992). However, it is also associated with a 25–50% incidence of grade 3+ toxicity. Some advantages of preoperative therapy have been noted, including increased resectability and increased chance of sphincter-sparing surgery. A retrospective comparison of two parallel phase I clinical studies was performed to compare preoperative and post-operative adjuvant treatment. In one trial (Minsky et al, 1991), 16 patients with unresectable rectal cancer received two cycles of bolus 5-fluorouracil (5-FU) (200–250 mg m$^{-2}$ with leucovorin 200 mg m$^{-2}$) plus radiation (total dose of 5040 cGy to the pelvis) before surgery, followed by a median two further cycles of chemotherapy after surgery. In the second trial (Minsky et al, 1992*b*), 25 patients with resectable disease were given the same regimen, but with all treatments being administered after surgery.

Despite significantly more patients in the preoperative group than in the post-operative group having received the higher dosage (250 mg m$^{-2}$) of 5-FU (75% vs 32%, $P = 0.02$), the overall incidence of acute grade 3+ toxicity was significantly higher in post-operative than in preoperative patients (Figure 1). The two grade 3 toxicities in the preoperative patient group were gastrointestinal in nature, whereas there were seven gastrointestinal and two genito-urinary toxicity reports, and four grade 4 toxicities, in the post-operative group. The data illustrate how the overall effectiveness of a treatment may be augmented to the patient's benefit through the design of a regimen that minimizes toxicity relative to other treatment schedules. They also demonstrate the importance of tolerability as an end point worthy of primary consideration by all those involved with the care of the patient.

A further example of how treatment can be manipulated to preserve the patient's quality of life is to be found in several series of data from non-randomized clinical studies in which therapies

**Figure 2** Effect of preoperative adjuvant radiotherapy (RT) or combined modality therapy (CMT) on patients with operable carcinoma of the rectum. Bars show the initial sample of patients who were judged clinically by the operating surgeon to require abdominoperineal resection (APR), and the proportions of these patients who underwent sphincter-sparing surgery subsequent to preoperative adjuvant therapy (Rouanet et al, 1995; Grann et al, 1997; Hyams et al, 1997; Maghfoor et al, 1997; Wagman et al, 1997)

© Cancer Research Campaign 1998

**Figure 3** A representation of patient-centred multidisciplinary management in advanced cancer were designed with the intention of preserving sphincter function. The standard surgical treatment for many distal rectal cancers is abdominoperineal resection (APR). This procedure involves the removal of the anal sphincter and therefore necessitates the formation of a permanent colostomy, with consequent deleterious effects on the patient's body function and image (Williams, 1984; Rouanet et al, 1993). The traditional rationale for this procedure lies in the natural history of these tumours, with their high risk of local recurrence (Domergue et al, 1988), the need for a 2-cm distal margin (Pollett and Nicholls, 1983; Williams et al, 1983) and the requirement for complete removal of the perirectal fat (McAnema et al, 1990; Leo et al, 1993). In recent years, improvements in surgery and the use of preoperative radiation therapy or combined modality therapy have allowed sphincter preservation in some patients who respond well to the down-staging effects of neoadjuvant therapies.

Data from five studies suggest that preoperative radiation therapy or combined modality therapy can benefit patients by making sphincter-preserving surgery possible (Rouanet et al, 1995; Grann et al, 1997; Hyams et al, 1997; Maghfoor et al, 1997; Wagman et al, 1997). Figure 2 shows patients who were able to undergo conservative surgery (local excision or a low anterior resection with or without a coloanal anastomosis) as a result of having received preoperative adjuvant radiation or combined modality therapy. In the majority of reports, sphincter-sparing surgery was made possible in 76–85% of patients who, on the basis of an examination, were judged clinically to require an APR with a permanent colostomy. Had adjuvant therapy not been made available to these patients, it is likely that all would have required an APR and associated permanent colostomy. Thus, these findings have considerable implications for the patient's quality of life, body image and psychological well-being. Furthermore, in the two series reporting sphincter function, it was reported to be 'good' or 'excellent' in 85% (Wagman et al, 1997) and 'perfect' in 71% (Rouanet et al, 1995) of evaluable patients. Although the phase I/II data that suggest that preoperative therapy has lower acute toxicity and enhances sphincter preservation need to be confirmed in a randomized trial, the results are nonetheless illustrative of quality-of-life end points.

### PATIENT CONSIDERATIONS AND SOCIAL, SOCIETAL AND COMMUNITY PERSPECTIVES

The global effect of treatment on the patient is of significant importance and consists of several overlapping aspects, including physical distress (e.g. adverse effects of treatment) and psychological stress (e.g. anxiety, depression and problems associated with body image) (Redmond, 1997). Considerations that are likely to be of immediate significance to the patient are those relating to toxicity and inconvenience of treatment, and loss of body function and image. Common toxicities associated with chemotherapy for advanced CRC include mucositis, diarrhoea, alopecia, nausea and vomiting, asthenia and hand–foot syndrome. Inconvenience is likely to be caused to the patient by frequent hospitalization, loss of work and family time, difficulties in travelling and compliance with some modes of administration of chemotherapy (e.g. continuous infusions of 5-FU). The financial difficulties related to hospital stays, high medication costs or loss of work that can be caused by cancer treatment are such that the appropriate measurement of costs incurred by the patients is an issue in itself in the economic evaluation of cancer treatment (Bonsel et al, 1993). Patients also experience the damaging psychological effects of loss of body image when faced with the possibility of loss of sphincter function in rectal cancer and the prospect of stoma care.

There are also social, societal and community-related perspectives to consider in anti-cancer therapy. These issues tend to be neglected by physicians and include the stresses on the patient as described earlier. Families and close friends may experience the negative aspects of cancer treatment, which include financial factors, social isolation and stress, in a similar way to the patient. These are all issues relevant to the discussion of quality-of-life end points and the overall implications of treatment of advanced CRC.

Anti-cancer treatments also have a major impact on medical and nursing workloads: two regimens might be similar in efficacy but might differ in their adverse effect profiles and complexity and frequency of delivery. Effective communication across medical, surgical and paramedical disciplines and with patients is needed to identify treatments that are more convenient to deliver and manage.

### A PATIENT-CENTRED MULTIDISCIPLINARY MANAGEMENT MODEL

Using the patient-centred multidisciplinary management model, the patient is considered as being at the centre of a continual process (patient-centred) that starts at diagnosis and ends, for all patients with advanced disease, in death. Components that feed into this therapeutic process are shown in Figure 3. General practitioners and patient support services are pictured against the patient to show their continuing presence and input from beginning to end. Other practitioners, including medical and radiation oncologists, surgeons and oncology nurses sometimes have contact with the patient for only relatively short and discrete periods after diagnosis, and there is therefore a tendency to neglect the need for continual and ongoing care and support. Ideally, all disciplines should operate together as a single unit, with the patient at the centre of the treatment process at all times.

This process benefits both patients and care providers, with the interprofessional exchange of information being of particular value to physicians who prescribe treatment. The economic, social and societal impact of each treatment should also be assessed. The multidisciplinary approach to the management of patients with advanced CRC addresses important treatment end points that tend to be overlooked, although they are, according to the patient-centred model, as important as survival. These include tolerability, quality of life and patient satisfaction. Thus, in the assessment of

© Cancer Research Campaign 1998

therapy for advanced CRC, patient satisfaction with treatment is a goal. There is a wide range of outcomes to be considered, and accurate quality-of-life information (if available) makes a major contribution to improving the management of these patients (Donovan et al, 1989). The IWGCRC has recommended the adoption of the multidisciplinary approach to deliver the best overall evaluations and subsequent outcomes for patients. Furthermore, it has identified the need for and the composition of the multidisciplinary team, has put forward international treatment recommendations and is currently discussing the development of tools to assist with the assessment of patient satisfaction.

### ACKNOWLEDGEMENTS

The International Working Group in Colorectal Cancer is supported by a grant from Zeneca Pharmaceuticals.

### REFERENCES

Bonsel GJ, Rutten FFH and Uyl-de-Groot CA (1993) Economic evaluation alongside cancer trials: methodological and practical aspects. *Eur J Cancer* **29A**(suppl. 7): S10–S14

Domergue J, Rouanet P, Daures JP, Kasse A, Dubois JB, Joyeux H, Solassol C and Pujol H (1988) Cancer du rectum: traitement par association radio chirurgicale de 238 malades. Etude mono et multifactorielle des facteurs de pronostic. *Gastroenterol Clin Biol* **12**: 797–802

Donovan K, Sanson-Fisher RW and Redman S (1989) Measuring quality of life in cancer patients. *J Clin Oncol* **7**: 959–968

Douglass HO, Moertel CG and Mayer RJ (1986) Survival after postoperative combination treatment of rectal cancer. *N Engl J Med* **315**: 1294–1295

Gastrointestinal Tumor Study Group (1985) Prolongation of the disease-free interval in surgically treated rectal carcinoma. *N Engl J Med* **312**: 1465–1472

Glimelius B, Hoffman K, Graf W, Haglund U, Nyrén O, Påhlman L and Sjödén P-O (1995) Cost-effectiveness of palliative chemotherapy in advanced gastrointestinal cancer. *Ann Oncol* **6**: 267–274

Grann A, Minsky BD, Cohen AM, Saltz L, Guíllem JG, Pary PB, Kelsen DP, Kemeny N, Ilson D and Bass-Loeb J (1997) Preliminary results of preoperative 5-fluorouracil, low-dose leucovorin, and concurrent radiation therapy for clinically resectable T3 rectal cancer. *Dis Colon Rectum* **40**: 515–522

Hyams DM, Mamounas EP, Petrelli N, Rockette H, Jones J, Wieand HS, Deutsch M, Wickerham DL, Fisher B and Wolmark N (1997) A clinical trial to evaluate the worth of preoperative multimodality therapy in patients with operable carcinoma of the rectum. *Dis Colon Rectum* **40**: 131–139

Krook JE, Moertel CG, Gunderson LL, Wieand HS, Collins RT, Beart RW, Kubista TP, Poon MA, Meyers WC and Mailliard JA (1991) Effective surgical adjuvant therapy for high-risk rectal carcinoma. *N Engl J Med* **324**: 709–715

Leo E, Belli F, Baldini MT, Vitellaro M, Santoro N, Mascheroni L, Andreola S, Bellomi M, Rebuffoni G and Zucali R (1993) Total rectal resection, colo-endoanal anastomosis and colic reservoir for cancer of the lower third of the rectum. *Eur J Surg Oncol* **19**: 283–293

Maghfoor I, Wilkes J, Kuvshinoff B, Westgate S, Bryer M, Perry MC, Miedema B, Doll D and Ota D (1997) Neoadjuvant chemoradiotherapy with sphincter-sparing surgery for low lying rectal cancer (abstract). *Proc Am Soc Clin Oncol* **16**: 274

McAnema OJ, Heald RJ and Lockhart-Mummery HE (1990) Operative and functional results of total mesorectal excision with ultra-low anterior resection in the management of carcinoma of the lower one-third of the rectum. *Surg Gynecol Obstet* **170**: 517–521

Minsky BD, Kemeny N, Cohen AM, Enker WE, Kelsen DP, Reichman B, Saltz L, Sigurdson ER and Frankel J (1991) Preoperative high-dose leucovorin/5-fluorouracil and radiation therapy for unresectable rectal cancer. *Cancer* **67**: 2859–2866

Minsky BD, Cohen AM, Kemeny N, Enker WE, Kelsen DP, Reichman B, Saltz L, Sigurdson ER and Frankel J (1992*a*) Combined modality therapy of rectal cancer: decreased acute toxicity with the preoperative approach. *J Clin Oncol* **10**: 1218–1224

Minsky B, Cohen A, Enker WE, Kelsen DP, Kemeny N, Riechman B, Saltz L, Sigurdson ER and Frankel J (1992*b*) Phase I trial of postoperative 5-FU, radiation therapy, and high dose leucovorin for resectable rectal cancer. *Int J Radiat Oncol Biol Phys* **22**: 139–145

Moinpour CM, Feigl P, Metch B, Hayden KA, Meyskens Jr FL and Crowley J (1989) Quality of life end points in cancer clinical trials: review and recommendations. *J Natl Cancer Inst* **81**: 485–495

Nordic Gastrointestinal Tumor Adjuvant Therapy Group (1992) Expectancy or primary chemotherapy in patients with advanced asymptomatic colorectal cancer: a randomized trial. *J Clin Oncol* **10**: 904–911

Palmer KR, Kerr M, Knowles G, Cull A, Carter DC and Leonard RC (1994) Chemotherapy prolongs survival in inoperable pancreatic carcinoma. *Br J Surg* **81**: 882–885

Pollett WG and Nicholls RJ (1983) The relationship between the extent of distal clearance and survival and local recurrence rates after curative anterior resection for carcinoma of the rectum. *Ann Surg* **198**: 159–163

Pyrhönen S, Kuitunen T and Kouri M (1992) A randomized, phase III trial comparing fluorouracil, epidoxorubicin and methotrexate (FEMtx) with best supportive care in non-resectable gastric cancer. *Ann Oncol* **3**(suppl. 5): 47

Redmond K (1997) The need for endpoints in anticancer drug trials that will simplify the clinical decision-making process. *Eur J Cancer* **33**(suppl. 2): S11–S13

Rouanet P, Saint Aubert B, Fabre JM, Astre C, Liu JZ, Dubois JB, Joyeux H, Solassol C and Pujol H (1993) Conservative treatment for low rectal carcinoma by local excision with or without radiotherapy. *Br J Surg* **80**: 1452–1456

Rouanet P, Fabre JM, Dubois JB, Dravet F, Saint Aubert B, Pradel J, Ychou M, Solassol C and Pujol H (1995) Conservative surgery for low rectal carcinoma after high-dose radiation. *Ann Surg* **221**: 67–73

Rougier P, Laplanche A, Huguier M, Hay JM, Ollivier JM, Escat J, Salmon R, Julien M, Roullet Audy JC and Gallot D (1992) Hepatic arterial infusion of floxouridine in patients in liver metastases from colorectal carcinoma: long-term results of a prospective randomized trial. *J Clin Oncol* **10**: 1112–1118

Scheithauer W, Rosen H, Kornek G-V, Sebasta C and Depisch D (1993) Randomised comparison of combination chemotherapy plus supportive care with supportive care alone in patients with metastatic colorectal cancer. *Br Med J* **306**: 752–755

Wagman R, Minsky BD, Cohen AM, Guillem JG and Paty PB (1997) Sphincter preservation with pre-operative radiation therapy (RT) and coloanal anastomosis long term follow-up. *Int J Radiol Oncol Biol Phys* **39**: 167

Williams NS (1984) The rationale for preservation of the anal sphincter in patients with low rectal cancer. *Br J Surg* **71**: 575–581

Williams NS, Dixon MF and Johnston D (1983) Reappraisal of the 5 cm rule of distal excision for carcinoma of the rectum: a study of distal intramural spread and of patient's survival. *Br J Surg* **70**: 150–154

 © Cancer Research Campaign 1998